# A Korean validation study of the Clinically Useful Anxiety Outcome Scale: Comorbidity and differentiation of anxiety and depressive disorders

**DOI:** 10.1371/journal.pone.0179247

**Published:** 2017-06-12

**Authors:** Sang Won Jeon, Changsu Han, Young-Hoon Ko, Seoyoung Yoon, Chi-Un Pae, Joonho Choi, Jae-Min Kim, Ho-Kyoung Yoon, Hoseon Lee, Ashwin A. Patkar, Mark Zimmerman

**Affiliations:** 1 Department of Psychiatry, Sungkyunkwan University School of Medicine, Kangbuk Samsung Hospital, Seoul, Republic of Korea; 2 Department of Psychiatry, College of Medicine, Korea University, Ansan Hospital, Ansan, Republic of Korea; 3 Department of Psychiatry, The Catholic University of Korea College of Medicine, Seoul, Republic of Korea; 4 Department of Psychiatry, College of Medicine, Hanyang University, Guri Hospital, Guri, Republic of Korea; 5 Department of Psychiatry, Chonnam National University Medical School, Gwangju, Republic of Korea; 6 Department of Psychiatry, St. Andrew’s Neuropsychiatric Hospital, Icheon, Republic of Korea; 7 Department of Psychiatry and Behavioral Sciences, Duke University Medical Center, Durham, North Carolina, United States of America; 8 Department of Psychiatry and Human Behavior, Brown University School of Medicine, Rhode Island Hospital, Providence, Rhode Island, United States of America; Taipei Veterans General Hospital, TAIWAN

## Abstract

**Background:**

This study aimed to evaluate the psychometric properties of the Korean version of the Clinically Useful Anxiety Outcome Scale (CUXOS) and to examine the current diagnostic comorbidity and differential severity of anxiety symptoms between major depressive disorder (MDD) and anxiety disorders.

**Methodology:**

In total, 838 psychiatric outpatients were analyzed at their intake appointment. Diagnostic characteristics were examined using the structured clinical interview from the DSM-IV because the DSM5 was not available at the start of the study. The CUXOS score was measured and compared with that of 3 clinician rating scales and 4 self-report scales.

**Principal findings:**

The CUXOS showed excellent results for internal consistency (Cronbach’s α = 0.90), test–retest reliability (*r* = 0.74), and discriminant and convergent validity. The CUXOS significantly discriminated between different levels of anxiety severity, and the measure was sensitive to change after treatment. Approximately 45% of patients with MDD were additionally diagnosed with anxiety disorders while 55% of patients with anxiety disorders additionally reported an MDD. There was a significant difference in CUXOS scores between diagnostic categories (MDD only, anxiety only, both disorders, and no MDD or anxiety disorder). The CUXOS scores differed significantly between all categories of depression (major, minor, and non-depression) except for the comparison between minor depression and non-depression groups.

**Conclusions:**

The Korean version of the CUXOS is a reliable and valid measure of the severity of anxiety symptoms. The use of the CUXOS could broaden the understanding of coexisting and differentiating characteristics of anxiety and depression.

## Introduction

Major depressive disorder (MDD) and anxiety disorders are highly prevalent and are often diagnosed together [1]. Half of the patients experiencing a new episode of MDD are known to satisfy the full DSM-IV criteria for current anxiety disorder [2]. If MDD and anxiety disorder co-exist, more characteristic clinical manifestations will appear than are observed with pure MDD. When anxiety symptoms are more severe, the severity and duration of the major depressive episode increases. Additionally, the response to depression treatment is less effective, remission is more difficult, and social dysfunction becomes more severe [3–5].

The knowledge of the presence of accompanying anxiety disorder in patients with MDD has very important treatment implications. First, awareness of the presence of a comorbid anxiety disorder influences the choice of medication prescribed. In general, the antidepressants with proven efficacy are known to have almost equivalent therapeutic effects on depression. The therapeutic effects of antidepressants in patients with anxiety disorders, however, may not be equivalent [6, 7].

Second, there is variability in the required medication dose and the side effects [8, 9]. In the clinical setting, when two disorders are comorbid, a higher dose of SSRI is often required for treatment [10]. In addition, initial antidepressant administration frequently worsens the symptoms of anxiety in patients with the two disorders, and such patients are more sensitive to the various side effects of antidepressant treatment [8, 11].

Third, the comorbidity of MDD and anxiety disorders affects the selection of the psychotherapy prescription. Various psychosocial interventions are known to be effective for specific anxiety disorders [12, 13]. Therefore, clinicians must select an appropriate psychotherapy for patients with concurrent anxiety disorder.

As we have seen above, determining whether an individual patient has depression, anxiety, or both is very useful for deciding a treatment method and monitoring the treatment process. In actual clinical practice, however, clinicians often preferentially concentrate their efforts on diagnosing MDD and measuring the severity of the depression due to limitations in human resources, time, and costs [10]. Consequently, either information regarding anxiety symptoms is indirectly obtained through a depression measurement scale, or assessment is often postponed until the patient returns for subsequent visits [14]. However, typical depression scales do not include all of the items related to anxiety symptoms and may not properly reflect the full dimensions of anxiety that changes during depression treatment [15].

The Clinically Useful Anxiety Outcome Scale (CUXOS) [16] is a brief and accurate self-report questionnaire used to evaluate the severity of anxiety frequently, quickly, and at minimal cost. The questionnaire takes two minutes to complete, and the completed form can be marked within 15 seconds. The CUXOS was developed based on the conventional clinician rating scale (the Hamilton Anxiety Rating Scale [HAM-A]) and the descriptions of panic disorder and generalized anxiety disorder in the DSM-III-R and DSM-IV. Although the CUXOS is not based on the current DSM5, the CUXOS is the most recent self-reported anxiety scale using the latest version of the DSM. Recent studies have shown that the CUXOS has very good compatibility with the DSM5 [17]. The CUXOS was originally developed to have sufficient critical scientific review and to allow other investigators to further examine its properties [16]. The reliability and validity of the CUXOS have been demonstrated through extensive patient samples [1, 16]. The CUXOS is focused on the general measurement of psychic and somatic anxiety rather than being an anxiety-specific scale, and it is useful for evaluating patients with depression and high levels of anxiety. This self-administered CUXOS includes items sufficient to measure somatic anxiety and is helpful in evaluating somatic symptoms accompanying anxiety disorder or MDD. The form consists of 20 self-administered questions: a 6-item psychic anxiety subscale and a 14-item somatic anxiety subscale. The respondents are asked to answer each question according to “how well it describes you during the past week, including today” using a 5-point Likert scale (0 = not at all true; 1 = rarely true; 2 = sometimes true; 3 = usually true; and 4 = almost always true). The CUXOS examines the respondent’s week prior to evaluation. The advantage of the CUXOS is that it can be used to systematically assess ongoing anxiety at subsequent visits.

The first objective in this study was to evaluate the psychometric properties of the Korean version of the CUXOS in a psychiatric outpatient sample and to further validate the existing English version of the measure. The second objective was to examine the current diagnostic comorbidity for patients between MDD and anxiety disorders. Lastly, we examined whether the anxiety severity indicated by CUXOS scores significantly differs between the DSM-IV diagnostic categories: 1) MDD only, anxiety disorder only, both MDD and anxiety disorder, and no MDD or anxiety disorder; 2) different depression diagnostic categories (major, minor, and non-depression); and 3) different anxiety disorders.

## Materials and methods

### Design and setting

A prospective and observational study was conducted in the typical clinical setting of outpatient psychiatric facilities. Clinical samples were collected at four hospital sites serving urban communities in South Korea. The study protocol was reviewed and approved by the institutional review board of the Korea University Ansan Hospital (IRB No. AS16106). Written informed consent was submitted by all participants upon enrollment.

### Participants

A total of 1,011 outpatients who satisfied the selection criteria were originally screened. The inclusion criteria were as follows: 1) new outpatients at the first psychiatric examination or patients who had not received antidepressant or other psychotropic drug treatment within the past 4 weeks and 2) patients aged 19 or older. The exclusion criteria were as follows: 1) patients who either were illiterate or had cognitive impairment that prevented them from answering the questions appropriately, 2) patients with current manic or psychotic symptoms, and 3) patients with underlying medical or surgical disorders that could affect the evaluation of the study. A total of 72 patients were excluded using these criteria, and 101 patients failed to complete all the measures. Thus, the final analyzed sample included 838 participants.

### Instruments

Anxiety symptoms were assessed using the CUXOS, two clinician rating scales (the HAM-A and the Clinical Global Impression for Severity [CGI-S]) and a self-report scale (the Beck Anxiety Inventory [BAI]). In this study, participants were rated on the Clinical Global Impression for Anxiety Severity (CGI-AS). Depressive symptoms were assessed using a clinician rating scale (the 17-item Hamilton Depression Rating Scale [HAMD]) and two self-report scales (the Patient Health Questionnaire-9 [PHQ-9] and the Beck Depression Inventory [BDI]). In addition, somatic symptoms were assessed using a self-report scale, the Patient Health Questionnaire-15 (PHQ-15). All administered measures in the Korean version were validated in previous studies [18–23].

### Linguistic adaptation of the CUXOS to Korean from the original version in English

Three board-certified psychiatrists and three certified psychologists who were fluent in both English and Korean translated the CUXOS into Korean and then back into English. Translation and back-translation of the CUXOS were repeated after state-of-the-art procedures in cross-cultural assessment were implemented [24]. The final version was reviewed by a professional translator and scholars of Korean literature and was approved by all the investigators.

### Analysis of the psychometric properties of the adapted version of the CUXOS

The interviews and tests were performed by 12 board-certified psychiatrists. They examined current diagnostic characteristics using the structured clinical interview for the DSM-IV (SCID) [25]. The participants completed the test before meeting the clinician and all psychiatrists were kept blinded to the participant’s responses on the questionnaire. Inter-rater reliability was examined in 16 patients and was found to be satisfactory by the HAMD (*r* = 0.95, p < 0.001), HAM-A (*r* = 0.64, p < 0.001), and CGI-AS (*r* = 0.78, p < 0.001). Of the 838 participants who carried out the CUXOS at the time of their intake appointment, 121 returned to the study center one week later and filled out the CUXOS a second time for test–retest reliability. Fifty-nine participants with anxiety disorder at baseline completed the CUXOS and were evaluated using the CGI-AS for a second time, 8 to 16 weeks after treatment, to investigate the sensitivity of the scale to changes in symptoms.

### Data analysis

The internal consistency was evaluated using Cronbach’s alpha and the item–total correlation. The test–retest reliability was assessed using the intra-class correlation coefficient (ICC). The concurrent and discriminant validity was assessed by correlating the CUXOS with the other measures of anxiety, depression, and somatic symptoms. All patients were classified and grouped according to MDD and anxiety disorder categories (MDD only, anxiety disorder only, both MDD and anxiety disorder, and no MDD or anxiety disorder) and DSM-IV depression categories (major depression, minor depression, and non-depression). The difference in the CUXOS scores between these classifications was evaluated through an analysis of variance (ANOVA) and post-hoc comparison of Tukey’s honestly significant difference (HSD) test. The CUXOS scores in participants with each of the DSM-IV anxiety disorders were compared with the scores in participants with no current anxiety disorder using *t*-tests. The ability of the CUXOS to discriminate between levels of anxiety severity was investigated based on CGI-AS rating. An ANOVA and post-hoc comparison of Tukey’s HSD were used. The sensitivity of the scale to changes after treatment was investigated based on the degree of improvement of CGI-AS rating using *t*-tests and paired *t*-tests. All the analyses were conducted using SPSS 20.0 for Windows. All the statistical tests were two-tailed. Statistical significance was set at p < 0.05.

## Results

### Sociodemographic and DSM-IV diagnostic characteristics

A total of 838 participants including 349 men (41.6%) and 489 women (58.4%) who met all the inclusion and exclusion criteria and completed all the examination and measures were analyzed in this study. The mean participant age was 43.9 ± 15.3 years (range = 20–76 years). The educational level of the participants was as follows: 0–6 years (37.0%, n = 310), 7–9 years (20.2%, n = 169), 10–12 years (30.9%, n = 259), or 13 years and above (11.9%, n = 100). Approximately two-fifths of the participants were married (43.7%, n = 366); the remainder were single (33.8%, n = 283) or divorced (22.6%, n = 189). The number of participants in each of the four sites was 344 (41.1%), 201 (24.0%), 167 (19.9%), and 126 (15.0%). There were no significant differences in sex, age, education level, and marital status among the four site groups.

The current DSM-IV Axis I diagnoses of participants at their intake appointment are given in Table 1. The most frequent DSM-IV diagnosis was MDD (41.2%, n = 345). The most frequent anxiety disorder was panic disorder (14.2%, n = 119). For all participants, the mean total score of the CUXOS was 27.3 ± 21.3.

**Table 1:** Current diagnostic characteristics of the participants (n = 838) by structured clinical interview for DSM-IV (SCID).

DSM-IV diagnosis	Frequency	Percentage
Major depression		
Major depressive disorder	345	41.2
Bipolar disorder (major depressive episode)	18	2.1
Minor depression		
Depressive disorder not otherwise specified	8	0.9
Adjustment disorder (with depressed mood)	27	3.2
Dysthymic disorder	70	8.3
Anxiety disorders		
Generalized anxiety disorder	60	7.2
Panic disorder	119	14.2
Social phobia	61	7.3
Specific phobia	7	0.8
Posttraumatic stress disorder	59	7.1
Acute stress disorder	8	0.9
Obsessive compulsive disorder	56	6.7
Other DSM-IV disorders		
Bipolar disorder (not major depressive episode)	55	6.6
Adjustment disorder (without depressed mood)	13	1.6
Alcohol abuse/dependence	57	6.8
Somatoform disorder	121	14.4
Schizophrenia	4	0.5
Other psychiatric disorder	86	10.3

Participants could be given more than one diagnosis. DSM-IV was used instead of the current DSM5 for diagnostic classification because only the DSM-IV was available at the start of the study.

### Reliability of the CUXOS

Cronbach’s α was 0.90 (*p* < 0.001) at the baseline. The item–total correlations ranged from 0.48 to 0.80 (mean = 0.663; all *p* < 0.001) at baseline. The test–retest reliability coefficient was 0.74 (*p* < 0.001).

### Discriminant and convergent validity of the CUXOS

Table 2 shows the significant positive correlations between the CUXOS and other measures (all *p* < 0.001). The CUXOS was more highly correlated with measures of anxiety (mean r = 0.740) than with measures of the other symptom domains (mean r = 0.532).

**Table 2:** Correlations between scores on the CUXOS and related measures.

		Anxiety				Depression			Somatic
		CUXOS	HAM-A	CGI-AS	BAI	HAMD	PHQ-9	BDI	PHQ-15
Anxiety	CUXOS	1.000							
	HAM-A	0.788	1.000						
	CGI-AS	0.714	0.844	1.000					
	BAI	0.718	0.751	0.812	1.000				
Depression	HAMD	0.510	0.377	0.510	0.559	1.000			
	PHQ-9	0.497	0.416	0.712	0.399	0.734	1		
	BDI	0.512	0.556	0.788	0.402	0.805	0.803	1.000	
Somatic	PHQ-15	0.610	0.565	0.873	0.470	0.670	0.647	0.587	1.000

All correlations are significant at p < 0.001; CUXOS: Clinically Useful Anxiety Outcome Scale; HAM-A: Hamilton Anxiety Rating Scale; CGI-AS: Clinical Global Impression for Anxiety Severity; BAI: Beck Anxiety Inventory; HAMD: Hamilton Depression Rating Scale; PHQ-9: Patient Health Questionnaire-9; BDI: Beck Depression Inventory; PHQ-15: Patient Health Questionnaire-15

### Prevalence and comorbidity of current MDD and anxiety disorders

**Fig 1:**
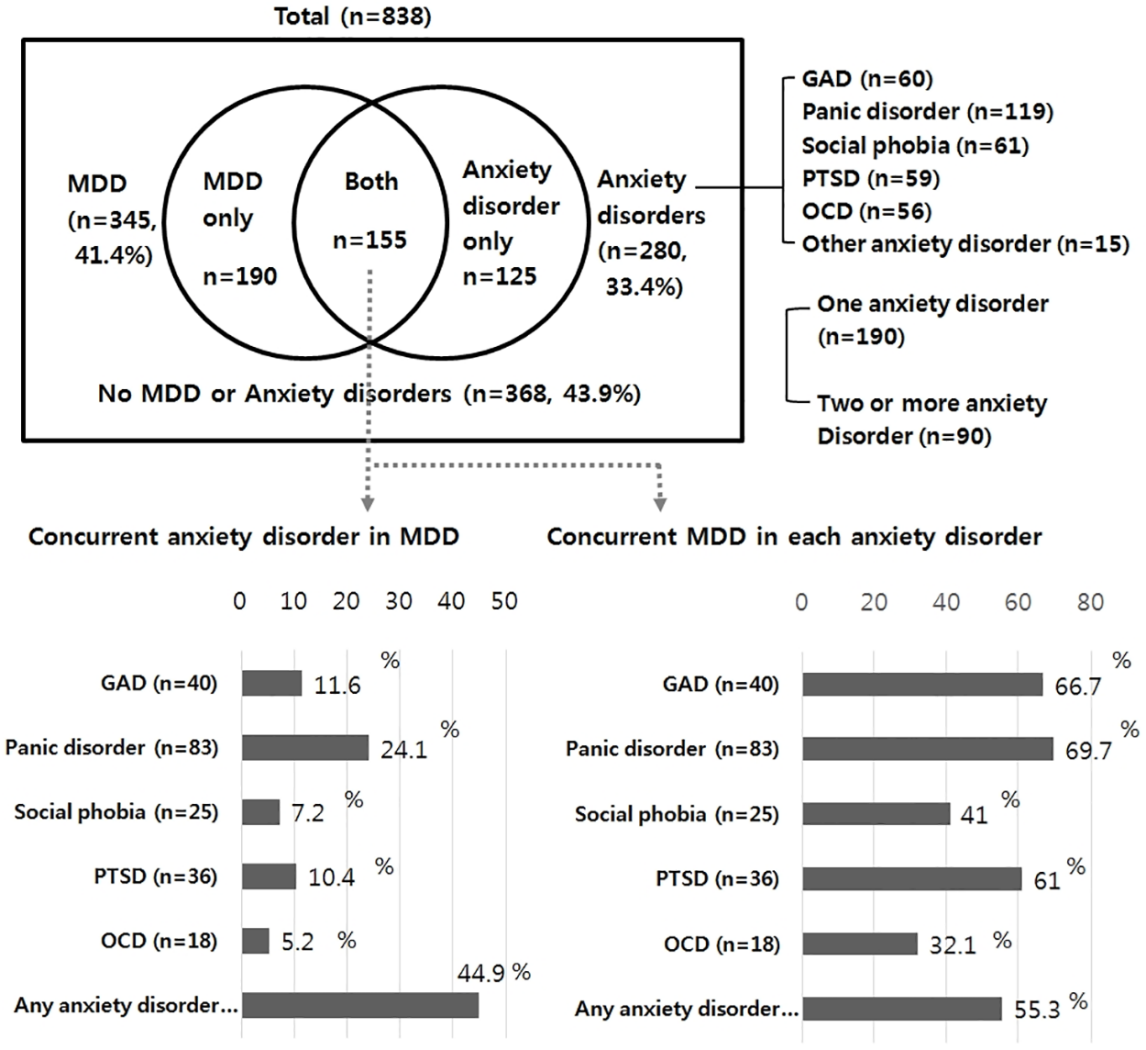
Diagnostic prevalence and differential comorbidity between current MDD and different anxiety disorders. MDD: major depressive disorder; GAD: generalized anxiety disorder; panic d/o: panic disorder; PTSD: posttraumatic stress disorder; OCD: obsessive compulsive disorder.

The prevalences of patients with a new episode of MDD, current anxiety disorders, and both conditions in psychiatric outpatient samples were 41.4%, 33.4% and 18.5%, respectively. Approximately 45% of patients with a new episode of MDD were additionally diagnosed with one or more anxiety disorders, and 55% of patients with a current anxiety disorder additionally reported an MDD. The various anxiety disorders also have high comorbidity. The anxiety disorder that most frequently co-occurred with MDD was panic disorder. The patients who were diagnosed with panic disorder, generalized anxiety disorder, and posttraumatic stress disorder were more likely to be co-diagnosed with MDD than those diagnosed with social phobia or obsessive-compulsive disorder (Fig 1).

### CUXOS total score comparison between MDD and anxiety disorders

We examined anxiety severities indicated by CUXOS scores in patients with MDD only, anxiety disorder only, both MDD and anxiety disorder, and no MDD or anxiety disorder. Table 3 shows the CUXOS and BDI scores of patients in each of these four diagnostic categories. There was a significant difference in the CUXOS (F = 129.4; *df* = 3,834; p < 0.001) and BDI scores (F = 185.4; *df* = 3,834; p < 0.001) between these diagnostic categories. Table 3 shows Tukey post-hoc comparisons between these diagnostic categories.

**Table 3:** CUXOS and BDI total score comparison between major depressive disorder and anxiety disorder.

Current diagnosis	MDD only	Mixed MDD and anxiety d/o	Anxiety d/o only	No MDD or anxiety d/o	Tukey post-hoc comparisons
Number of subjects	190	155	125	368	
%	27.0	14.2	19.2	43.9	
CUXOS mean (SD)	25.9 (13.2)	45.8 (18.1)	34.1 (16.4)	22.6 (15.2)	All p < 0.001 except for MDD only and no MDD or anxiety d/o (p = 0.08)
BDI mean (SD)	29.4 (15.4)	44.5 (18.2)	16.4 (10.1)	10.5 (6.7)	All p < 0.001 except for anxiety d/o only and no MDD or anxiety d/o (p = 0.02)

MDD: major depressive disorder; Anxiety d/o: anxiety disorder; CUXOS: Clinically Useful Anxiety Outcome Scale; BDI: Beck Depression Inventory

### CUXOS total score comparison between the depression diagnostic categories

We examined the CUXOS score in patients diagnosed with each of the DSM-IV mood disorders. The majority of patients with each mood disorder were diagnosed with MDD. The remaining mood disorders made up a relatively small sample size. Patients with mood disorder were grouped and classified into major depression, minor depression, and non-depression groups. The major depression group consisted of 363 participants diagnosed with MDD (41.2%, n = 345) or a bipolar major depressive episode (2.1%, n = 18). The minor depression group consisted of 106 participants who were not diagnosed with major depression but were diagnosed with a depressive disorder not otherwise specified (0.9%, n = 8), an adjustment disorder with depressed mood (3.1%, n = 28), or a dysthymic disorder (8.3%, n = 70). The non-depression group consisted of 397 participants who were not diagnosed with major depression or minor depression.

The mean BDI scores of the major depression group, minor depression group, and non-depression group were 32.5 ± 15.7, 23.3 ± 12.7, and 10.5 ± 6.68, respectively. The three-group ANOVA was significant (F = 60.7; *df* = 2,835; p < 0.001), and the differences among the three groups were significant using Tukey’s HSD test.

The mean CUXOS scores of the major depression, minor depression, non-depression groups were 34.1 ± 10.8, 26.3 ± 19.7, and 24.0 ± 18.8, respectively. The three-group ANOVA was significant (F = 29.8; *df* = 2,835; p < 0.001). Tukey’s test showed that the between-group differences were significant except for the comparison between minor depression and non-depression group (p = 0.26).

### Comparison of CUXOS scores between the DSM-IV anxiety disorders

Table 4 shows the CUXOS scores of participants with each of the DSM-IV anxiety disorders. Each of these CUXOS scores was compared with the scores of 488 patients with no current anxiety disorder. Participants with each of DSM-IV anxiety disorders received significantly higher CUXOS scores than participants with non-comorbid anxiety disorders (all p < 0.001; Table 4).

**Table 4:** The CUXOS scores of participants with and without DSM-IV anxiety disorder.

Current anxiety disorder	Mean CUXOS Score (SD)	T	p
Panic disorder (n = 119)	46.3 (21.2)	16.7	< 0.001
Generalized anxiety disorder (n = 60)	37.2 (20.2)	12.4	< 0.001
Social phobia (n = 61)	29.8 (20.1)	9.1	< 0.001
Obsessive-compulsive disorder (n = 56)	35.2 (21.2)	7.2	< 0.001
Posttraumatic stress disorder (n = 59)	42.1 (18.3)	10.2	< 0.001
Any anxiety disorder (n = 280)	33.5 (20.4)	12.9	< 0.001
No current anxiety disorder (n = 488)	17.9 (15.3)		

The CUXOS scores in participants with each of the DSM-IV anxiety disorders were compared with the scores in participants with non-comorbid anxiety disorder.

The patients diagnosed with only one anxiety disorder (n = 180) had higher CUXOS scores than those with two or more anxiety disorders (n = 90). The respective CUXOS scores were 31.2 ± 17.6 and 39.2 ± 24.2 (*t* = 14.5, p < 0.001).

### The ability of the CUXOS to discriminate between levels of anxiety severity

The ability of the CUXOS to discriminate between different levels of anxiety severity in all participants was investigated with an ANOVA based on the CGI-AS ratings. Because the participants rated on CGI 1 (n = 15) or CGI 7 (n = 10) were considered to be a relatively small sample size, the two lowest (CGI 1, 2) and two highest (CGI 6, 7) rating levels were grouped into single categories. The total CUXOS scores increased with increases in the CGI-AS score (CGI 1–2 [n = 147]: 14.2 ± 8.9; CGI 3 [n = 154]: 20.7 ± 8.1; CGI 4 [n = 213]: 28.1 ± 10.9; CGI 5 [n = 216]: 37.2 ± 11.2; CGI 6–7 [n = 108]: 48.5 ± 14.3). The five-group ANOVA was significant (F = 82.2, *df =* 4,833, p < 0.001). Tukey’s test showed that the differences between each adjacent CGI-AS level were significant except for the comparison between participants with CGI 1–2 and CGI 3 (*p* = 0.07).

We repeated the above analysis, limiting the sample to the 350 patients diagnosed with one or more anxiety disorder. The range of CGI-AS scores was truncated, and all but 44 patients (from 306 participants) were rated CGI 3 (22.1 ± 10.7), CGI 4 (27.6 ± 8.7), and CGI 5 (37.0 ± 9.4). The three-group ANOVA was significant (F = 33.8, *df =* 2,303, P < 0.001), although the variability in the CGI scores was reduced. The differences between the three groups were significant using Tukey’s HSD test.

### Sensitivity to change in symptoms after treatment

Fifty-nine participants with panic disorder (n = 37) or generalized anxiety disorder (n = 22) at baseline completed the CUXOS and were evaluated using CGI-AS at a second visit 8 to 16 weeks after treatment. Participants were classified into responder (n = 40) and non-responder groups (n = 19). The responder group contained the participants with two or more steps of improvement on the CGI-AS rating (for example, CGI from 5 to 3, CGI from 5 to 2). The non-responder group contained the participants with a maximum of one step improvement on the CGI-AS rating (for example, CGI from 4 to 3, CGI from 3 to 3). There were no differences in the mean CUXOS scores between responder and non-responder group at baseline (37.5 ± 15.1 vs. 34.3 ± 16.3, *t* = 0.7, not significant). At the follow-up, the CUXOS scores of the responder group were significantly decreased (37.5 ± 15.1 vs. 18.4 ± 13.6, paired *t* = 4.1, p < 0.001). However, the scores of the non-responder group did not significantly change (paired t = 0.8, not significant). At the follow-up, the CUXOS scores were significantly lower in responders compared to those of non-responders (18.4 ± 13.6 vs. 31.8 ± 15.1, t = −2.4, p < 0.001).

## Discussion

The results of this study suggest that the Korean version of the CUXOS is a reliable and valid measure of the severity of anxiety symptoms. Consistent with the initial validation study of the English language version of the scale, internal consistency was high, all item–scale correlations were significant, and test–retest reliability was high. Moreover, the ability of the CUXOS to discriminate among different levels of anxiety severity was significant, and the measure was sensitive to change after treatment.

The depression and anxiety scales, including CUXOS, showed high correlations with one another (Table 2). This result supports the clinical experience that depression and anxiety have many common symptoms. Previous studies have argued that it is difficult to distinguish MDD from anxiety disorder based on depression and anxiety scores obtained with a self-reporting scale. It has been reported that the anxiety scores of patients with MDD were similar to or higher than those of patients with generalized anxiety disorder (GAD) or panic disorder and that the depression scores of the patients with anxiety disorder were reported to be higher than moderate [26–28]. First, this might have resulted from the diagnostic classification not using SCID. Second, the reason for such an occurrence might be that the previous studies did not consider the effect of comorbidity and did not classify the patients into those with pure anxiety disorder or pure MDD and those with the two concurrent disorders. In the present study, however, the CUXOS was more highly correlated with other measures of anxiety than with measures of other symptom domains, such as depression or somatic symptoms (Table 2). Additionally, the CUXOS significantly classified pure MDD, pure anxiety disorder, and both disorders (Table 3). The present study thus supports the presence of common symptoms between depression and anxiety as well as unique symptoms that can be differentiated and that belong to different dimensions of these disorders.

Previous hypotheses used to explain the coexisting and differentiating characteristics of anxiety and depression are primarily classified into two categories [29]: The dimensional approach and the syndromal approach. The dimensional approach [26, 30] claims that anxiety and depression are distributed along a single dimension with anxiety on one end and depression on the other. In this model, the center point is where anxiety and depression coexist. That is, it considers the symptom co-occurrence at the symptomatic level. For example, the patient’s state is described as “MDD with high levels of anxiety.” The syndromal approach [31, 32] diagnoses anxiety disorder and depressive disorder based on certain criteria, such as DSM, and deals with the comorbidity of these two disorders, the comorbidity rate, etc. In other words, it considers diagnostic comorbidity depending on the diagnostic level. For example, a patient’s state is described as “MDD with comorbid/concurrent anxiety disorder.” This syndromal approach has been applied by most of the previous studies and is often used in actual clinical practice. The approach has shortcomings, however; for example, patients whose anxiety symptoms are not sufficient to be diagnosed as anxiety disorder but are clinically important can be misdiagnosed [33].

In the diagnosis of MDD, if the comorbidity pattern of anxiety and depression is taken into account based on the diagnostic criteria, MDD can be classified into the following three cases [33, 34]: 1) one where MDD and anxiety disorder are both clearly diagnosed (co-existing MDD and anxiety disorder), 2) one accompanied by anxiety satisfying the diagnostic criteria of MDD but not satisfying the diagnostic criteria of anxiety disorder (MDD + subsyndromal anxiety disorder), and 3) one where the symptoms do not satisfy the diagnostic criteria of either MDD or anxiety disorder (subsyndromal depression and anxiety disorder). To make up for the shortcomings of MDD classification using this syndromal approach, scales should be used to measure the severity of symptoms at the subsyndromal level.

In this study, the self-reporting tests, CUXOS and BDI, were both able to accurately distinguish pure MDD, pure anxiety disorder, and both disorders (Table 3). Regarding the CUXOS score, the patients with pure anxiety disorder had significantly higher scores than those with pure MDD. Regarding the BDI score, the patients with pure MDD had significantly higher scores than those with pure anxiety disorder. This means that MDD can be significantly distinguished from anxiety disorder using a self-reporting test if an accurate diagnosis of either MDD or anxiety disorder is made and diagnostic comorbidity is considered. This implies that systematic diagnostic classification using a structured interview may help clinicians in their evaluation of patients complaining of depression or anxiety.

Previous studies using the CUXOS presented the score for each of the five categories of anxiety symptoms severity [16]. A total score of 0–10 is considered indicative of non-anxious state, 11–20 indicates minimal anxiety, 21–30 indicates mild anxiety, 31–44 indicates moderate anxiety, and 45 or higher indicates severe anxiety. In this study, the total mean CUXOS scores based on the CGI-AS rating (CGI 1–2: 14.2 ± 8.9, CGI 3: 20.7 ± 8.1, CGI 4: 28.1 ± 10.9, CGI 5: 37.2 ± 11.2, CGI 6–7: 48.5 ± 14.3) were thought to be consistent with the five categories of anxiety severity from previous study. Using these categories, pure MDD, pure anxiety, and both disorders fall under the mild, moderate, and severe anxiety levels, respectively. In this study, patients with both MDD and anxiety disorders showed significantly higher severity of depression and anxiety. Comorbidity of these two disorders was very significant with diagnosis of comorbidity in 53% of the patients with MDD and in 44.9% of the patients with anxiety disorder (Fig 1). For this patient group, careful frequent-measurement-based care must be given.

Non-depression, minor depression, and major depression fell under the mild, mild, and moderate anxiety levels, respectively. The differences in the BDI score among these three groups were significant, but in terms of the CUXOS, the score was high only in the major-depression group, and there was no difference in CUXOS scores between the minor-depression and non-depression groups. This means that the anxiety severity of MDD is noticeable among various mood disorders, and it is most important to measure the anxiety symptoms of MDD among the various mood disorders. According to previous studies, half of the patients with current anxiety disorder are known to have been diagnosed with more than two anxiety disorders simultaneously [1]. In this study, about one-third of the patients diagnosed with anxiety disorder had more than two anxiety disorders. Patients with multiple anxiety disorders showed a significant increase in the severity of anxiety compared with patients with one anxiety disorder.

Among those with anxiety disorder in this study, the CUXOS total score was highest in patients diagnosed with panic disorder and lowest in patients diagnosed with social phobia. Although the CUXOS has excellent ability to discriminate anxiety severity, a lower score for one anxiety disorder, such as social phobia, does not indicate a lower level of anxiety severity. A lower total score on the CUXOS does not suggest a minor form of anxiety disorder. The reason for this may be that the CUXOS was developed based on the DSM-III-R and DSM-IV diagnostic criteria of panic disorder and GAD [17]. In other words, the CUXOS is a measure of panic or general anxiety symptomatology rather than of anxiety in a specific condition such as social anxiety. Another desired purpose of using the CUXOS is to accurately measure the anxiety level of patients with MDD as well as of patients with a specific anxiety disorder. Therefore, the scale must be able to measure all aspects of anxiety and overall anxiety even though the validity for discriminating anxiety disorder may be somewhat sacrificed. For patients with a specific anxiety disorder, a scale specialized for each disorder must be used additionally. Moreover, it must be realized that the CUXOS scores of patients with MDD considerably reflect panic or GAD symptomatology, and additional measurement must be made depending on the characteristics of the patient’s anxiety.

### Limitations

Below are the points that must be considered in the interpretation of the results and limitations of this study: 1) as the DSM-IV was used, the results of this study may not be fully generalized in the current DSM5 practice environment. For example, because OCD and posttraumatic stress disorder were included as an anxiety disorder in our study, comorbidity of anxiety disorders and MDD may be different in DSM5 practice. This was inevitable for the consistency of this study because the DSM-IV was used at the starting point of the study. However, the DSM5 also focuses on symptom classification rather than cause of mental disorders. Therefore, no matter what version of the DSM is used, the findings of the distinction and commonality between depression and anxiety disorders still have their important clinical significance. 2) This study focuses on a clinical setting and will be more meaningful to clinicians. However, an important limitation of this study is that the participants included were restricted to clinical psychiatric outpatients. The participants had a high proportion of depression, and the sample size of older adults was relatively small because many elderly participants were excluded based on the exclusion criteria. This sample may not be fully representative of community-dwelling populations or the primary care setting. The generalizability to participants with different socio-demographic (e.g., elderly patients) or clinical characteristics (e.g., medical/surgical patient setting) will need to be validated. 3) This study targeted only new outpatients at their intake appointment and, thus, did not show the lifetime prevalence of the disorders but only suggested the prevalence of the current or concurrent disorder. In previous studies, the overall rate of the comorbidity of the current anxiety disorder with depression (44.7%) was only slightly lower than the lifetime comorbidity (46%; 36). 4) As the sample sizes rated for CGI 1 or CGI 7 were considered relatively small, five categories of CGI-DS instead of seven were explored to identify the ability to discriminate between the levels of severity. 5) The depression diagnostic category used in this study is not a category that is commonly used in other studies. It is a category we suggest be used in investigating the severity of anxiety in various mood disorders as well as MDD.

## Conclusions

The results of this study reveal that the Korean version of the CUXOS has good reliability and validity. In conclusion, the CUXOS is a very useful scale for screening for anxiety symptoms and following up on the progress of anxiety disorders. Additionally, it supplements the clinical diagnosis of depression by enabling clinicians to systematically evaluate comorbid anxiety using a standardized instrument.

In actual clinical practice, the systemic assessment of comorbid anxiety disorders or symptoms in patients with MDD may not be easy. It is very important to accurately diagnose MDD and anxiety disorder, identify diagnostic comorbidity, and clearly measure the severity of anxiety in patients with MDD. This is because awareness of specific comorbid anxiety disorders or anxiety symptoms in patients with MDD may influence therapeutic decisions, particularly the focus of psychosocial intervention, such as CBT. The use of clinical assessment such as the CUXOS could broaden the understanding of common and unique dimensions of depression and anxiety and may provide many applications for clinical treatment.
